# Healing Presence: an Intensive Care Unit Curriculum for Medical Students Based on the Clinical Pastoral Education Training Model

**DOI:** 10.1111/tct.70389

**Published:** 2026-03-25

**Authors:** S. Anderson, L. J. Brazier, J. Baruch, C. Wright, K. Collier

**Affiliations:** ^1^ School of Psychology Eastern Michigan University Ypsilanti Michigan USA; ^2^ Department of Spiritual Care University of Michigan Ann Arbor Michigan USA; ^3^ Department of Psychiatry University of Michigan Ann Arbor Michigan USA; ^4^ Department of Internal Medicine University of Michigan Ann Arbor Michigan USA

## Abstract

**Background:**

In the United States, approximately 90% of the population believes in God or a higher power. Spirituality is integral to decision‐making for some patients, and when spiritual needs are met, healthcare costs may be reduced. At the University of Michigan Medical School, we observed a paucity of education in spiritual care. We developed an elective to engage medical students in reflecting on spirituality in the care of critically ill patients and their own experiences and evaluated the impact of this educational model.

**Approach:**

Senior medical students shadowed chaplains and participated in reflection sessions with chaplain‐led peer groups. A retrospective pre–post survey was collected from students to assess the impact of the elective.

**Evaluation:**

Seventy‐eight students participated in the elective between 2017 and 2020. After the elective, students reported increases in how much they value finding personal meaning in patient care (88%– 100%) and responding to patients' spiritual concerns (53%–94%). Review of narrative feedback revealed that the elective fostered a more inclusive understanding of spirituality, heightened recognition of spiritual care as an essential yet often overlooked aspect of care, increased awareness and empowerment to use available resources, and meaningful personal and professional growth within a safe, reflective space.

**Implications:**

The elective shifted student perception on the importance of the role of spirituality in medicine. Educational initiatives such as this help address a need within undergraduate medical education and can serve as a platform for integrating spirituality and interprofessional education and collaboration into medical training.

## 1 Background

Spirituality includes the ways that individuals relate to themselves, to others, to the world around them, to the significant or sacred and how they seek and express meaning and purpose [1]. Spirituality, therefore, is often part of the frameworks through which patients and healthcare professionals alike understand and make sense of the things that happen while seeking out healthcare and practicing healthcare as a profession.

Several regulatory and accreditation bodies have formally recognised the relationships between spirituality and health in both medical education and professional practice. The Joint Commission on the Accreditation of Healthcare Organizations outlines requirements for adequate spiritual care to be provided by hospitals [2]. The Liaison Committee on Medical Education (LCME) requires medical schools to teach students to recognise beliefs and values that inform how people interpret health, illness and how people respond to various symptoms, diseases and treatments [3]. The Association of American Medical Colleges (AAMC) recommends medical students be educated in a core set of spiritual competencies. In addition, they list spiritual self‐awareness for medical students as a core spiritual competency [4].

However, research demonstrates gaps in healthcare providers' abilities to integrate these competencies clinically. Healthcare providers report inconsistent abilities to recognise the spiritual needs of patients, despite research showing that patients want these needs addressed [5, 6, 7]. This suggests that adequate training is lacking and further education on this topic is needed [8]. The training to address spiritual competencies also varies with minimal clinical integration. One survey of deans from 122 US medical schools found that 90% reported having an elective course or didactic content related to spirituality and health. However, 90% of those electives or content were provided in the preclinical year [9]. Spirituality is also related to how physicians and medical students seek and express meaning in their work, which has been shown to be protective against burnout for physicians [10]. Therefore, medical students need opportunities to develop awareness of their spiritualities as well as others'.

*Healthcare providers report inconsistent abilities to recognise the spiritual needs of patients, despite research showing that patients want these needs addressed*.

## 2 Approach

We sought to address this need and design an elective that could occur during students' clinical years. Medical students were invited to opt into the Healing Presence elective while on their intensive care unit sub‐internship rotation. The nongraded elective was taught by two chaplains in the Department of Spiritual Care at Michigan Medicine. Both chaplains have significant training and experience in healthcare chaplaincy including teaching and specialised training in specialty clinical areas.

Learning objectives outlined for the elective included (1) providing students with a framework to reflect on emotions involved in caring for critically ill and dying patients, (2) reflecting on the interpersonal dynamics of patient care, (3) developing primary spiritual care skills particularly spiritual history taking and (4) exploring how students' values and beliefs influence their approach to patient care or challenges with medical training.

During the elective, students shadowed a chaplain and engaged in four semi‐structured, 1‐h small group reflective sessions facilitated by a chaplain. Shadowing included observation and debriefing of clinical encounters. Students were also given prompts to reflect on clinical encounters on the unit during their rotation. Each prompt was an invitation for students to notice their own responses to a clinical situation, patient, family, or other provider(s) or to notice situations and people that are ‘sticking with them’. Students were invited to share these reflections in the small group sessions.

The first session of the small group focused on defining spirituality, spiritual needs related to health and students' own spiritual histories. Sessions 2 and 3 focused on spiritual reflection: Students were invited to present a case that either made them feel uncomfortable (values dissonance), that gave them a sense of fondness for the patient or family (value‐congruence) or a spiritual history that they took with a patient or family member. Together, students discussed, in a supportive learning environment, key aspects of the encounter along with questions for reflection. Instruction was provided by the chaplain instructor as needed, based on themes that arose from these discussions. Session 4 readdressed questions asked at the first session to reflect on how students' perspectives may have changed during the elective. The fourth session also reinforced instruction that was most relevant to each group, such as trauma‐informed care, individuals' routines and rituals, or post‐traumatic growth.

## 3 Evaluation

Pre–post surveys were sent to 78 former and current senior medical students from our academic medical centre who completed the Healing Presence elective from 2017 to 2020. Participation was fully voluntary, and no incentives were offered. Of the 78 students who completed the elective, 35 students responded and completed the survey. The survey showed the greatest changes in reported attitudes related to taking spiritual histories, chaplain involvement if the student or their family member were hospitalised, and creating treatment plans that involve chaplains (see Table 1). Narrative comments qualified these themes, as well as finding value in small group reflective discussions (see Table 2).

**TABLE 1:** Average reporting of student perception regarding the importance of spirituality for themselves and for patients.

Question	Pre (average)	Post (average)	Sum difference	% increase
Inquiring about patients' spiritual and religious beliefs	3.14	4.11	0.97	30.89
Having chaplaincy involved if you or a family member were hospitalised	3.09	4.34	1.25	40.45
Creating treatment plans that include chaplaincy resources	2.83	4.03	1.2	42.40
Responding to patients' spiritual concerns	3.71	4.63	0.92	24.79
Taking spiritual histories from patients	2.63	3.94	1.31	49.81
Being aware of your own well‐being for the sake of patient care	3.86	4.63	0.77	19.95
Finding personal meaning in taking care of patients	4.43	4.8	0.37	8.35
The interpersonal components of patient care	4.51	4.8	0.29	6.43
Considering how your own spiritual well‐being affects your patient care	3.14	4.23	1.09	34.71

**TABLE 2:** Narrative feedback.

What was the greatest insight you had with this program?	‘Recognizing that spirituality is an entirely separate entity from religion, and that failing to acknowledge a patient's faith or recognize spiritual distress can adversely affect their well‐being and health’.
	‘I assumed the chaplaincy service were only for highly religious Christian patients, but I learned that they work with patients from all walks of life and see everyone as a ‘spiritual being’.
What was the most impactful part of the healing presence program for you?	‘Selfishly, it was our therapeutic debriefing sessions that helped me the most where we talked about the conversations we'd seen, the events we'd witnessed and what insight or truth to pull from them’.
	‘I really appreciated having a safe space to discuss what I was struggling with during this rotation’.
How did you grow from the healing presence program?	‘I did a spiritual history on my own which was a really meaningful and powerful learning experience, and plan to do this with future patients as well. I also reflected on my own spirituality and the meaning I find in working with patients, which was very important for my own mental health’.
	‘More aware of resources available to me as staff. Never really thought of utilizing a chaplain or social worker when I'm dealing with difficult situations such as patient death’.
	‘Talking through patient cases that were challenging to me helped me reflect on the biases I bring to patient care as a provider’.
How do you think this program was relevant to your medical training?	‘It was eye‐opening and made me realize how underutilized the chaplaincy program is’.
	‘Got me back in touch with why I became a physician. Helped embolden me to act based on my values and sense of purpose in the hospital’.
	‘I think this program is very relevant. Spirituality is intermingled with the mind and body. It is an important aspect of patient care that is neglected. This program sets aside time for us to think about how we might approach this topic with patients that is natural for us’.
	‘I felt that many patients might have spiritual crises when in the ICU, and I wanted to feel more empowered to address and respond to those concerns’.

Prior to taking the elective, only 6/35 (17%) students believed that taking spiritual histories was ‘important’ or ‘very important’. After the elective, this increased to 26/35 (74%). One student commented, ‘I did a spiritual history on my own which was a really meaningful and powerful learning experience, and plan to do this with future patients as well’. Another student shared, ‘[spirituality] is an important aspect of patient care that is neglected. This program sets aside time for us to think about how we might approach this topic with patients that is natural for us’.

Similar in the pre‐elective section, 10/35 (29%) students rated chaplaincy involvement as important or higher; post‐elective, this increased to 30/35 (86%). One student reported, ‘I assumed the chaplaincy service were only for highly religious Christian patients, but I learned that they work with patients from all walks of life and see everyone as a “spiritual being”’.

Relatedly, only 5/35 (23%) students believed that creating treatment plans that included spiritual care was either important or very important prior to this elective. Afterward, however, 29/35 (83%) students rated it as important or higher post‐elective.

Another change in reported attitudes was in finding personal meaning in taking care of patients (4.43 pre‐elective to 4.8 post‐elective) and in the interpersonal components of patient care (4.51 pre‐elective to 4.8 post‐elective). A student shared, ‘[The elective] Got me back in touch with why I became a physician. Helped embolden me to act based on my values and sense of purpose in the hospital’.

Several respondents also shared they valued the opportunities to reflect on their own emotions, beliefs and values related to clinical experiences in the ICU. One student wrote, ‘Talking through patient cases that were challenging to me helped me reflect on the biases I bring to patient care as a provider’ and another shared, ‘… it was our therapeutic debriefing sessions that helped me the most where we talked about the conversations we'd seen, the events we'd witnessed and what insight or truth to pull from them’. Another respondent wrote, ‘I really appreciated having a safe space to discuss what I was struggling with during this rotation’.

## 4 Implications

Our survey suggests that this elective helped equip students to discuss patients' spiritual histories and to collaborate with chaplains. Students also developed a greater appreciation of the roles of spirituality and spiritual care in medicine and a greater appreciation for taking time to reflect on their own beliefs, values, sense of meaning, and purpose.

Furthermore, because some of the respondents completed the survey up to 3 years after taking the elective, we can infer some durability of these lessons.

The outcomes of this survey suggest that our elective is one model that can help fulfil LCME and AAMC requirements related to spirituality and serve as a framework for future courses, rotations and training in primary spiritual care for students and providers. This elective provides a relatively low‐cost model for student education that could be replicated in any medical school that has a well‐staffed Department of Spiritual Care. Future efforts to more fully evaluate the impact of the elective would be beneficial.

## 5 Limitations

This elective takes place in a large academic medical centre, with an average chaplain‐bed ratio, an accredited Clinical Pastoral Education (CPE) program and a few highly specialised chaplains who have experience with clinical teaching. While most hospitals [11] in the United States do have some kind of spiritual care provider on staff, not all chaplains on staff may have capacities to take on these educational roles.

Our survey was given post‐elective, using a retrospective pre–post survey method, and 35/78 (44%) participants responded. As such, there is likely selection bias in these results. Additionally, narrative feedback was analysed descriptively rather than through a formal qualitative methodology, limiting the depth and transferability of these findings.

## Author Contributions

**Steven Anderson:** writing – original draft, writing – review and editing. **LJ Brazier:** writing – review and editing, conceptualisation, resources. **Jeremy Baruch:** writing – review and editing, conceptualisation, resources. **Christina Wright:** writing – review and editing, conceptualisation, resources. **Kristin Collier:** project administration, conceptualisation, writing – review and editing, supervision.

## Funding

The authors have nothing to report.

## Ethics Statement

This initiative did not receive funding institutionally or with grants. The authors included in this manuscript have no conflict of interest. Patient interaction was performed under standards of clinical care. Additionally, this was an educational initiative within the School of Medicine and is not involved in a clinical trial. Given this, the initiative did not require IRB approval.

## Conflicts of Interest

The authors declare no conflicts of interest.

## Data Availability

The data that support the findings of this study are available on request from the corresponding author. The data are not publicly available due to privacy or ethical restrictions.
